# Short-term visits by eye care professionals: ensuring greater benefit to the host community

**Published:** 2008-12

**Authors:** Andy Pyott

**Affiliations:** Consultant Ophthalmologist; Medical Advisor CBM; Raigmore Hospital, Old Perth Road, Inverness IV2 3UJ, UK.

**Figure:**
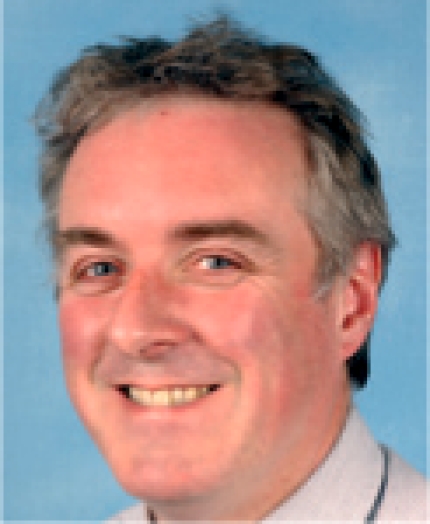


In ophthalmology, even brief encounters with patients can have a dramatic effect on their health and quality of life, whether this is through sight-restoring surgery or corrective spectacles.

The relative affluence of medical staff in some countries and the availability of cheap international fights means that it is easy for these doctors, nurses, orthoptists, etc. to take leave and parachute themselves into another country for a week or two of eye care. A recent article in *BMJ Careers* directs the interested to a range of nongovernmental development organisations who might be able to facilitate the adventure.^1^

I would like to question whether such expeditions achieve anything more than mere ‘ophthalmic tourism’. How can we ensure that the greater benefit is to the host community?

The purpose of this article is not to discourage involvement in cross-cultural projects, but rather to help enthusiastic ophthalmology volunteers from all countries to channel their energy in the most productive manner.

## Pitfalls of cross-continental exchanges

### Unfamiliar clinical territory

Part of the joy of travel is the encounter with the unfamiliar, but consequently it is possible to make assumptions about medical practice which do not transfer to another context. The first visit anywhere will almost certainly involve mistakes being made.

The disease profiles of patients show geographical variation and ophthalmologists from temperate climates are less familiar with common tropical conditions, such as fungal keratitis. Furthermore, the management strategy for a corneal ulcer in their own country may be very different to that in the country being visited.

### Donations of consumables

Unfamiliarity is even more critical in the operating theatre. In most wealthy countries, practice relies extensively on disposable items. The necessity to reuse in poorer countries may be a problem for the visitor. If the visitor is profligate with precious supplies, this may also be a problem for the host!

**Figure:**
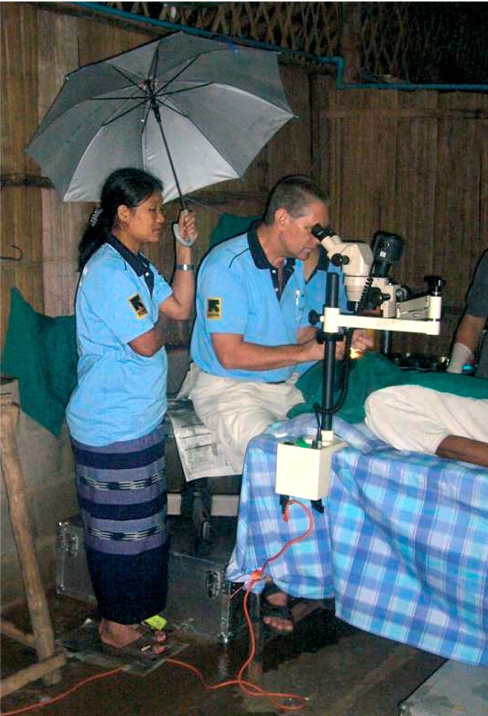
Operating under a leaky roof in the Karen community. THAILAND

Many visitors, however, arrive with charitable supplies of equipment and consumables. It is essential that such donations are appropriate. Drug companies may be keen to clear unused stock nearing expiry, writing it off against tax. There is the risk that supplies are merely transferred from one stockroom to another, often leaving the donor more satisfied than the recipient. For example, the only benefit of a recent donation of oxygen concentrators to a hospital in The Gambia was the establishment of a committee to oversee the acceptance of unsolicited donations.^2^

### Unfamiliar surgical techniques

A number of years ago, I conducted an anonymous survey of young doctors in an Asian country as to the benefits of visits from foreign eye surgeons. They cited ‘donated consumables’ as being the most important. This was ironic, as the visitors' remits were usually to train and mentor newly qualified doctors. In many cases, the visits were unsuccessful because the visitors were not familiar with the surgical techniques being used. Later audit revealed that sometimes the visiting trainers' surgical outcomes were worse than those of their trainees.

Training in Western countries gives little exposure to suturing techniques and there is an enthusiasm for junior doctors to travel to developing countries to gain experience in surgery they would not get at home. Where this is part of an established exchange of skills, with appropriate supervision, this is beneficial to both parties. However, any suggestion that junior doctors may go to another country and practice, on disadvantaged people, techniques they would not be permitted to use in their own countries is immoral and unethical.

### Destabilising the local eye health services

All development assistance should be established around the principle of local needs assessment and requests for help. Volunteer health programmes have a curious knack of being clustered around popular tourist destinations, and whilst permission may have been sought from officials in the ministries of health (who might have their own agendas), sometimes the involvement of local ophthalmologists is not sought.

This can be very destabilising. Offering free care to poor people seems such an obvious solution when viewed from afar, but if it upsets the local economy and jeopardises a cost-recovery scheme at a nearby hospital, it may ultimately cause far more problems than it solves.

For example, cataract being a chronic disability, relatives may defer using services at the local clinic, if waiting another year means that their grandmother will get the surgery free from an exotic foreigner. Not only will this be detrimental to the local clinic, but if the grandmother's deteriorating vision is actually caused by glaucoma, the delay may also lead to permanent and irreversible blindness.

### Free services?

All cultures have a fascination with the stranger beyond the borders, and the unscrupulous may see this as a means of generating some income. Thus, whilst the visitor may be donating his or her services for free, some may view the foreign expert as a marketable commodity. There may be hidden payoffs, financial, political, or religious, of which the visiting eye professional will be unaware.

### Phacoemulsification versus SICS: responsible skills transfer

The local needs assessment is likely to establish that skills transfer is what is desired. This may well include a request for training in phacoemulsification.

It is interesting to speculate whether phacoemulsification would have become so dominant in the wealthier nations if small incision cataract surgery (SICS) had been developed first. Studies^3^,^4^,^5^ have indeed demonstrated that the surgical outcomes of the two techniques can be similar, and that SICS is faster, cheaper, and less technology-dependent than phacoemulsification. Not all UK ophthalmologists are aware of this.

Of course, phacoemulsification can play a part in blindness reduction programmes, and many cataract surgeons want to have a broad range of skills to offer their patients.

Whilst I do know of circumstances where surgeons have become competent in phacoemulsification following two weeks of intensive training, this is not what we would expect of trainees in the UK or North America. Delivering a less thorough training in a potentially dangerous and highly expensive technique may not be in the best interest of a country's blindness prevention programme. An important principle to bear in mind is that all surgeons should be using “the right operation, for the right patient, at the right time!”

If training in phacoemulsification is part of a planned visit, both sides must ensure that the equipment is available, is of an adequate standard, and can be maintained when it breaks down. In addition, suitable arrangements must be made for the treatment of patients who suffer complications, such as a retained nucleus fragment.

**Figure:**
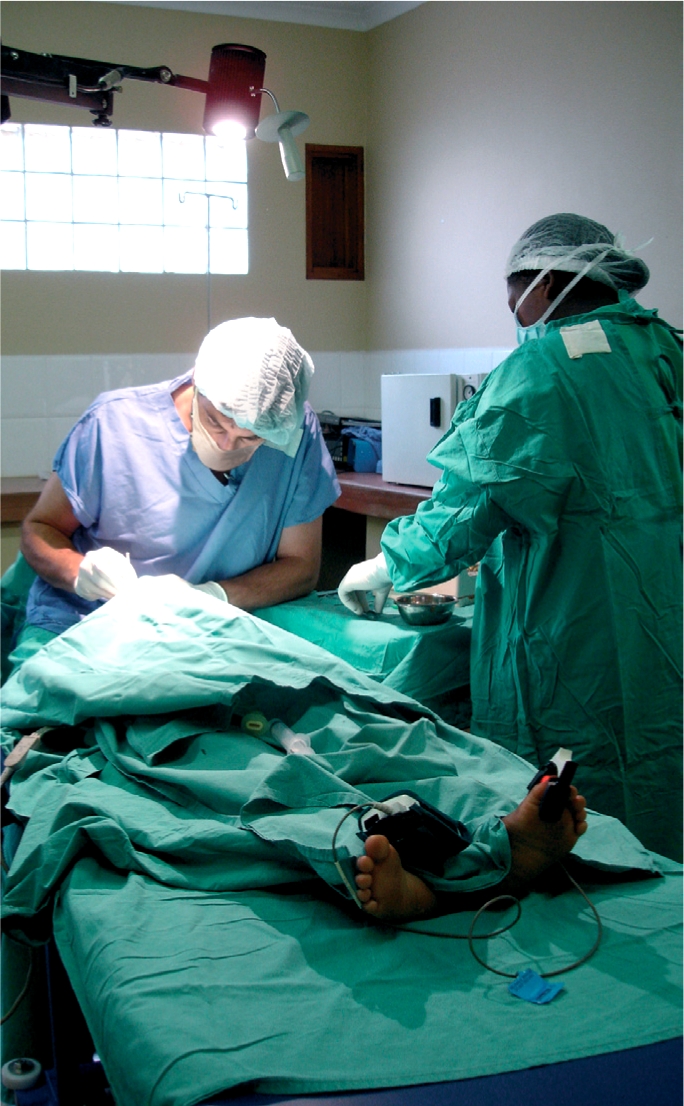
Training in paediatric surgery in the southern African region. SWAZILAND

## Maximising benefit to the local community

The Box on the right details important principles to maximise the benefits of a visit for the local community. If these principles are ignored, a visit can easily create more problems than it can solve. There are many examples of successful cross-continental visits and exchanges. The following selection provides an illustration of these principles.

### Providing care to isolated and disadvantaged communities

Many isolated communities have no hope of an ophthalmic service, but for the intervention of an outsider.

The Vine Trust^6^ set up a floating medical clinic on the Amazon River, which provides a service to remote and disadvantaged people and fulfils needs that would otherwise not be met.

Often, for small and isolated communities, visits from eye care staff will be the only stimulus for local services to be developed.

For example, Frank Green, a consultant ophthalmologist at Aberdeen University, has effectively been the sole ophthalmologist to the Karen refugees who have fled from Myanmar (Burma) to Thailand. Since 1990, he has made two to three visits each year. For political reasons, the Karen cannot receive ophthalmic medical support from their Thai hosts. The size of the community is sufficiently small to make it difficult to support and sustain a full-time ophthalmologist. However, over the years, a number of medical assistants have been trained locally to identify cataract and manage non-surgical cases.

### Providing expert training to meet local needs

In the 1980s, strabismus surgeons at Colombo Eye Hospital in Sri Lanka identified the need for orthoptic skills. However, sending staff to the UK to obtain orthoptic qualifications failed to provide a continuous service. With the backing of CBM initially, and later of the World Health Organization and of Sightsavers International, orthoptist Rowena McNamara was recruited for three months to design and deliver an orthoptic module, to add onto the ophthalmic technology course, with a curriculum to meet local needs. This has resulted in 20 years of uninterrupted orthoptic service at Colombo Eye Hospital provided by three full-time orthoptists. In 2000, a new clinic was started at Kandy Eye Hospital.

Another successful example of skills transfer meeting local needs is the formation of a vitreoretinal service at the Kikuyu Eye Unit, in Kenya. This was only possible as a result of regular visits by UK surgeons to East Africa, at the demand of local staff. A further consequence of this initial experience has been a commitment by the Jules Gonin Club to support vitreoretinal training in five institutions in low- and middle-income countries.

## Conclusion

The best interactions are those that enable long-term relationships and foster a genuine sharing of experiences and ideas. Successful exchanges work both ways: visitors will recognise that they are liable to gain more than they give. For example, a report to the UK government^7^ clearly acknowledged that the country benefits when its health care workers spend a period working overseas.

To facilitate such programmes, and to avoid some of the problems listed above, the International Centre for Eye Health (ICEH) advocates a proper needs analysis and an exchange through the Links Programme.^8^ This programme is proving to be extremely successful, matching recognised needs and the skills offered, and it is certainly the best way forward to maximise the global enthusiasm for involvement in VISION 2020.

**Principles for a beneficial visit by eye care staff**

It is crucial to consider all the following principles before embarking on a project:

1. **Response to local needs or request for help**
2. **Long-term commitment**
3. **Repeated visits**
4. **Full involvement of the local community and authorities**
5. **Commitment to the transfer of skills**
6. **Use of appropriate technology** (including surgical techniques and materials)
7. **Fostering a genuine sharing of experience and ideas**

**What do you think? Call for Exchange articles**

We look forward to receiving your suggestions on **how local staff can maximise the benefits of a visit from a foreign eye care team**, in the form of Exchange articles (500 words). Email: Anita.Shah@Lshtm.ac.uk (Subject: Foreign eye care teams).
